# Assessing the psychometric properties of the French WHOQOL-HIV BREF within the ANRS CO3 Aquitaine Cohort’s QuAliV ancillary study

**DOI:** 10.1186/s12955-020-01451-8

**Published:** 2020-07-10

**Authors:** Diana Barger, Mojgan Hessamfar, Didier Neau, Marc-Olivier Vareil, Estibaliz Lazaro, Pierre Duffau, Nicolas Rouanes, Olivier Leleux, Fabien Le Marec, Marie Erramouspe, Linda Wittkop, François Dabis, Fabrice Bonnet

**Affiliations:** 1 Univ Bordeaux, ISPED, Inserm Bordeaux Population Health, team MORPH3EUS, UMR 1219, CIC-EC 1401, F-33000 Bordeaux, France; 2 Services de Médecine Interne et Maladies Infectieuses, Centre Hospitalier Universitaire de Bordeaux (CHU), F-33000 Bordeaux, France; 3 COREVIH Nouvelle Aquitaine, Bordeaux, France; 4 Service de Maladies Infectieuses et Tropicales, Centre Hospitalier Universitaire de Bordeaux (CHU), F-33000 Bordeaux, France; 5 Service de maladies infectieuses, Centre Hospitalier de la Côte Basque, F-64100 Bayonne, France; 6 UMR-5164 CNRS, CIRID, University of Bordeaux, F-33000 Bordeaux, France; 7 Centre Hospitalier de de Périgueux, F-24000 Périgueux, France; 8 AIDES Nouvelle Aquitaine, Bordeaux, France; 9 Pôle de Santé Publique, Centre Hospitalier Universitaire de Bordeaux (CHU), F-33000 Bordeaux, France

**Keywords:** HIV, Health-related quality of life, WHOQOL-HIV BREF

## Abstract

**Background:**

Antiretroviral therapy has prolonged the lives of those with human immunodeficiency virus (HIV), but the effects of chronic infection on their health-related quality of life (HRQoL) remain a concern. Numerous instruments have been developed to measure HRQoL, yet evidence of their cross-cultural equivalence and continued applicability is limited. We adapted the WHOQOL-HIV BREF to French and assessed its psychometric properties in a sample of community-dwelling adults living with HIV who were mostly virally suppressed.

**Methods:**

We conducted a cross-sectional study within the ANRS CO3 Aquitaine cohort from July 2018 to May 2019. Five hundred eighty-six participants were consecutively enrolled at their HIV-consultations and completed either a web-based (*n* = 406) or paper self-administered assessment (*n* = 180). The means and standard deviations for items and domains were computed and the presence of floor and ceiling effects assessed. We evaluated internal consistency by calculating Cronbach’s alpha coefficients per domain. We assessed construct validity by performing a Confirmatory Factor Analysis (CFA). Concurrent, convergent and discriminant validity were assessed with Pearson’s correlations and known-group validity was assessed according to CD4 cell count, viral load, Centers for Disease Control and Prevention clinical categories for HIV, and hospitalization of more than 48 h within 2 years of the most recent consultation using one-way analysis of variance and independent *t*-tests.

**Results:**

Five hundred eighty-six PLWH were included in this analysis. Their median age was 55; 73% were male; 85% were of French descent; 99% were on ART and 93% were virally suppressed. We found floor effects for one and ceiling effects for 11 items. Four of the six domains showed good internal consistency (α range: 0.63–0.79). CFA showed that the WHOQOL-HIV BREF’s six-domain structure produced an acceptable fit (SRMR = 0.059; CFI = 0.834; RMSEA = 0.07; 90% CI: 0.06–0.08). It showed good concurrent, convergent and discriminant validity. There was some evidence of known-group validity. The personal beliefs domain had the highest score (15.04 ± 3.35) and the psychological health domain had the lowest (13.70 ± 2.78).

**Conclusions:**

The French WHOQOL-HIV BREF has acceptable measurement properties. Its broad conceptualisation of HRQoL, going beyond physical and mental health, may be of particular value in our older, treatment-experienced and virally suppressed population.

**Trial registration:**

ClinicalTrials.gov NCT03296202 (Archived by WebCite at http://www.webcitation.org/6zgOBArps).

## Background

Human immunodeficiency virus (HIV), once terminal, is now a manageable chronic illness [1]. Early and sustained access to antiretroviral therapy (ART) has reduced the risk of AIDS-related and non-AIDS-related events and has enabled people living with HIV (PLWH) to achieve normal life expectancies [2, 3]. Yet, the burden of HIV and associated inflammation, ART exposure, modifiable risk factors for age-associated conditions, other viral co-infections, and social and economic vulnerability make PLWH’s quality of life (QoL), both health-related and global, an ongoing concern [4]. In countries and regions where most PLWH are diagnosed, linked to care and have sustained access to effective ART, there have been resounding calls to “go beyond viral suppression”, and more specifically, to formally consider “good health-related quality of life (HRQoL)” as the ultimate metric of health system performance [5]. This has prompted a renewed interest in and demand for instruments to assess (HR)QoL in this patient population [6].

QoL has been defined by the World Health Organisation (WHO) as “an individual’s perception of their position in life in the context of the culture and value systems in which they live and in relation to their goals, expectations, standards and concerns” [7]. As many have found this definition too nebulous, the concept of HRQoL has been proposed, reflecting “the patient’s perception of the effect of illness and treatment on physical, psychological and social aspects of life” [8]. Several instruments have been developed to measure HRQoL in PLWH [9]. In a recent systematic review of reviews, Cooper et al. catalogued instruments, both generic and disease-specific, used to measure HRQoL in PLWH. They identified nine generic and seven disease-specific instruments that were comprehensive (covering at least three domains), could be self-administered in 10 min or less, and had been developed with input from PLWH [10]. The WHOQOL-HIV BREF and the PROQOL-HIV [11] were considered to have “promising psychometric properties and be more relevant to PLWH compared to MOS-HIV” [12], which has the most well-established psychometric properties but limited cross-cultural relevance and continued applicability [10]. Cross-cultural relevance is a concern as the majority of instruments were developed in North America, often with limited input from PLWH. Continued applicability has also been questioned owing to the relatively rapid evolution in the treatment of PLWH. Historically, PLWH were treated with ART based on clinical indications, such as opportunistic infections or CD4 cell count, long considered the primary laboratory indictor of immune function and a strong predictor of HIV progression [13]. France's current clinical guidance, which became effective in 2013 , calls for all PLWH to be offered ART, irrespective of CD4 cell count [14]. As nearly all disease-specific HRQoL instruments were developed before the current clinical guidance came into effect, ensuring their continued applicability has become increasingly important as we strive to move towards more person-centred HIV care. We therefore need to further valid HRQoL instruments in new populations and longitudinally.

The WHOQOL-HIV BREF, the short form of the WHOQOL-HIV [15, 16], was developed simultaneously within seven countries, allowing for better semantic and conceptual equivalence across cultures [17]. It covers six domains: (i) physical, (ii) psychological, (iii) level of independence, (iv) social, (v) environmental and (vi) spiritual QoL. The first four domains are likely directly affected by health and the use of medicines, whereas the last two domains (environmental and spiritual QoL), although important, may not be as frequently affected by healthcare. O’Connell and Skevington reported acceptable internal consistency (α = 0.69 for the spiritual - α = 0.82 for environmental QoL). They also reported that adding the HIV-specific items improved internal consistency [17]. Evidence from subsequent studies in Chinese and Malay populations have suggested good test-retest reliability [18, 19]. We opted to adapt the WHOQOL-HIV BREF for many of the reasons put forth by Cooper et al. [10]. First, it was created more recently than other disease-specific instruments, many of which were either developed prior to or shortly after effective ART [12]. Second, it was developed simultaneously in six culturally-diverse countries, making its cross-cultural equivalence potentially superior to instruments developed in a single population [17]. Third, the majority of items (26 out of 31) were generic as they stemmed from the WHOQOL-BREF instrument (derived from the WHOQOL-100) [20]. This was relevant given conclusions of previous studies of HRQoL in PLWH, stating that poorer HRQoL may in part be due to factors other than HIV infection [21].

### Aim

We aimed to adapt the English version of the WHOQOL-HIV BREF instrument to French and evaluate its psychometric properties in a population of older, treatment-experienced and mostly virally suppressed PLWH in Nouvelle Aquitaine, France to ensure both cross-cultural relevance and continued applicability [17].

## Methods

### Study population and procedures

The ANRS CO3 Aquitaine cohort is an open, prospective longitudinal study of adults (≥ 18 years old) with a confirmed HIV-1 diagnosis in care in 13 public hospitals in the Nouvelle Aquitaine region of south-western France. Experienced Clinical Research Associates extract epidemiological, clinical and laboratory data from patients’ medical records and enter them in a web-based electronic Case Report Form called ARPEGE 1.0. The QuAliV study is a cross-sectional survey conducted within the ANRS CO3 Aquitaine cohort. It aims to evaluate (HR)QoL and other patient-reported outcomes in PLWH in the current treatment era. The QuAliV study relies on a novel module designed for the collection of electronic patient-reported outcome (ePROs), including (HR)QoL. As described in detail elsewhere [22], the content of the ePROs module is based on current French treatment guidelines and is comprised of validated questionnaires, selected based on their established measurement properties and pragmatic considerations (e.g. self-administration, length etc.). Paper-versions of questionnaires were adapted to a screen format following the International Society for Quality of Life Research’s recommendations [23]. Before launching the pilot study, empirical, task-based usability evaluations were conducted on two successively developed prototypes of the ePROs module [24].

Cohort participants seen at participating clinical sites for their routine hospital-based HIV consultation were invited by investigators to join the QuAliV ancillary study. Investigators verified whether theoretically eligible participants were able to complete a self-administered assessment in French. Those who expressed interest were invited to complete the assessment online, provided they had a personal e-mail account and reliable Internet access. Participants were then issued a study-specific unique identifier, which enabled them to create an account independently and gain access to a secure web-based ePROs module to complete the assessment. An identical paper questionnaire was given to those who did not meet the basic requirements of the ePROs module. Participants either completed the paper questionnaire immediately or mailed it back to the hospital.

To form the French WHOQOL-HIV BREF, we used translations of items from the validated French WHOQOL-BREF [25] and the validated French WHOQOL-HIV [26]. As per O’Connell and Skevington’s original research article [17], HIV-specific items were: “How much are you bothered by any physical problems related to your HIV infection?”; “To what extent do you feel accepted by the people you know?”; “To what extent are you bothered by people blaming you for your HIV status?”; “How much do you fear the future?”; “How much do you worry about death?”. Cognitive debriefing was performed with native-speakers to ensure that items had good face validity (Supplementary Material 1).

### Data sources and variables

This analysis covers the period of the initial 10-months of implementation (July 23, 2018 – June 4, 2019) in five clinics located in Bordeaux (*n* = 3), Bayonne (*n* = 1), and Périgueux (*n* = 1). Participants consulting between July 23, 2018 – May 15, 2019 and invited to participate were considered for this analysis if they had provided informed consent and had at least one recorded hospital consultation or hospitalization between the 1st of January 2017 and the 6th of June 2019. All available self-reported data, saved as participants progressed through each stage of the assessment, were considered for analysis, regardless of whether or not they had been submitted. Paper questionnaires, returned prior to the 4th of June 2019, were entered and considered for this analysis.

Participants completed a self-administered questionnaire, reporting their educational attainment (ranging from none to 5 years post-secondary education or higher), net household income (ranging from less than 900€ to more than 4000€ per month), profession, employment status, and whether or not they lived with a partner. They also completed the French version of the WHOQOL-HIV BREF. Participants’ self-reported data were merged with those routinely collected from their medical records at either enrolment or at the most recent hospital consultation. We derived the participant’s age, transmission route (coded as men who have sex with men (MSM), heterosexuals, intravenous (IV) drug use, or other), place of origin, time in years since HIV diagnosis, time in years since start of first ART, HIV stage according to the Centers for Disease Control and Prevention (CDC) categories, and history of hospitalizations of > 48 h in the past 2 years [27]. Participants’ most recent CD4 cell counts (cells/mm^3^) and viral load (copies/mL) were considered for this analysis if they were recorded within a three-year window of the most recent consultation. CD4 cell counts were categorised according to the following clinically meaningful thresholds: < 200, 200–499, and ≥ 500 cells/mm^3^. Viral load measures are presented according to the following thresholds: < 50, 50–200, > 200 copies/mL or as less than or greater than 50 copies/mL.

### Sample size

As we intended to perform a confirmatory factor analysis (CFA), we followed Kline’s guidance, which is among the most conservative, regarding the required sample size. Kline recommends 10 to 20 observations per estimated parameter, where the number of identifiable parameters is, for the simplest of models, k items, *N*_*p*_ *= k x (k + 1)/2* [28]. We assumed that k equals 29 rather than 31 as two general items measuring overall quality of life and general health perception are not used to calculate the six domain scores. *N*_*p*_ therefore equals 435.

### Statistical analysis

All analyses were performed using STATA 15.1 (StatCorp LLC). Participants’ sociodemographic and HIV-related characteristics are described. Frequencies and proportions are presented for categorical variables and medians and interquartile ranges are presented for continuous variables.

The WHOQOL-HIV BREF is a 31-item self-reported questionnaire covering six domains with 29 items: physical (4 items), psychological (5 items), level of independence (4 items), social relationships (4 items), environmental (8 items) and spiritual (4 items) and two general items that measure overall quality of life and general health perception. Each item is rated on a 5-point Likert scale, where 1 denotes poor and 5 excellent. To obtain individual domain scores, negatively phrased items are reverse scored. The domain scores are then calculated by multiplying the mean of all items within the domain by 4. This results in six domain scores, each ranging from 4 (worst) to 20 (best). The six domain scores were calculated for those with complete data. We computed the proportion of missing responses for each item, omitting the first two items as these were compulsory in the questionnaire. We also computed the mean, standard deviation (SD), skewness, kurtosis, floor and ceiling effects of each item and domain. We assumed that there was a floor or ceiling effect if more than 20% of responses were in extreme categories (either 1 or 5).

We evaluated internal consistency, the extent to which the items are inter-related, for each domain using Cronbach’s alpha. Nunnally and Bernstein have proposed thresholds of 0.70–0.90 as a measure of good internal consistency [29]. The WHOQOL-HIV BREF’s concurrent validity was examined using Pearson’s correlations between domains and general quality of life (item 1) and health perception (item 2). We considered Pearson’s correlation coefficients to be weak (*r* < 0.3), moderate (r ≥ 0.3   < 0.7), or strong (*r* ≥ 0.7) and *p*-values of < 0.05 to be statistically significant. To test construct validity, we explored correlation patterns by constructing a correlation matrix between all pairs of items, using the hypothesized scale structure (Supplementary Material 2), and subsequently performed a CFA based on the original six-domain structure and assessed the pattern of item-domain relationships (factor loadings). It has been recommended that items with low factor loadings (e.g. below 0.2 or 0.3) be removed from the instrument [30]. We assessed goodness-of-fit using the approximate goodness-of-fit indices rather than the chi-square goodness-of-fit test based on Fayers and Machin’s recommendations [28]. We presented the Standardised Root Mean Square Residual (SRMR), the Comparative Fix Index (CFI) and the Root Mean Square Error of Approximation (RMSEA) as per Hu and Bentler’s guidance [31]. The proposed threshold for the SRMR is < 0.08. For the CFI, values > 0.95 are commonly used to indicate good fit and values of > 0.90 indicate acceptable fit; for the RMSEA, < 0.05 is considered excellent fit whereas 0.08 is considered acceptable fit. We present a path diagram of the postulated structure of the WHOQOL-HIV BREF instrument. We then examined modification indices and added error covariances between facets within the same domain in an effort to improve model fit. We assessed convergent and discriminant validity by calculating item-domain Pearson’s correlations. A correlation coefficient > 0.4 for items and their respective domains was considered to be satisfactory of convergent validity. Items revealing correlations with their respective domains that were higher than those with other domains were used to indicate good discriminant validity [28].

Known-group validity or the ability of the instrument to discriminate between specified groups of patients was assessed according to participants’ immunological (CD4 cell count) and virological status (viral load copies/mL). We hypothesized that participants with higher CD4 cell counts, indicating a stronger immune system, would have better HRQoL. We expected a CD4 cell count ≥500 cells/mm3 to correlate with higher mean domain scores. Conversely, we expected those with a detectable viral load, defined as > 50 copies/mL, to have poorer HRQoL. These hypotheses were tested using one-way analyses of variance (ANOVA) and independent sample *t-*tests. We also repeated analyses conducted by O’Connell and Skevington exploring mean differences in domain scores according to clinical categories for HIV infection as defined by the CDC’s 1993 Revised Classification System for HIV using ANOVA [17, 27]. We expected that those classified as clinical category A, reflecting asymptomatic HIV infection, would have higher HRQoL scores compared to those in clinical category B, which reflects HIV infection with symptoms directly attributable to HIV infection, or category C, which reflects those who have been diagnosed with AIDS. We further assessed known-group validity using evidence of hospitalization > 48 h within 2 years of the most recent consultation. We tested the null hypothesis of no difference in mean domain scores for those who had been hospitalized compared to those who had not been hospitalized within a two-year window of the most recent consultation using an independent *t*-test.

## Results

### Basic characteristics

The WHOQOL-HIV BREF questionnaire was completed by 587 PLWH during the study period. One observation was excluded due to delays in data entry. Five hundred eighty-six participants having completed at least the first item of the WHOQOL-HIV BREF were therefore considered for this analysis; 406 (69.3%) completed an electronic version of the questionnaire and 180 (30.7%) an identical paper version. Five hundred seventy-four participants had completed all items for physical health, 569 for psychological health, 560 for level of independence, 557 for social relations, 557 for environmental health and 570 for personal beliefs domains. The study population’s characteristics are described in detail in Table 1. Respondents were mostly male (*n* = 430, 73.2%) and their median age was 55 years old (IQR 48.9, 62.8). Eighty-five percent were of French descent. Forty-two percent (*n* = 248) reported living with a partner. The main transmission group was MSM (*n* = 290, 49.5%). The median time since HIV diagnosis was 20.1 years (IQR, 11.8, 27.7). Participants were treatment-experienced, with a median time since first ART of 16.4 years (IQR 8.3, 21.7). The vast majority (92.7%) were virally-suppressed (< 50 copies/mL). 117 (20.1%) had been diagnosed with AIDS (CDC Clinical Category C) and 31 (5.3%) had been hospitalized for > 48 h within the last 2 years since the most recent consultation. Compared to those actively followed up in the open centers, those who completed the assessment were slightly older (55 versus 53 years old), more often of French descent (85% versus 80%) and more likely to be MSM (49% versus 43%).

**Table 1:** Sociodemographic characteristics & HIV characteristics of the study population^a^ (*N* = 586)

	N	
Median age (IQR)	586	55.8 (48.9–62.8)
Sex (N, % male)	586	430 (73,2)
Level of education (%)		
None	40	6.8
Primary education	43	7.4
Secondary education	19	3.3
Vocational training	140	23.9
High school education	132	22.5
Associates	72	12.2
Undergraduate	62	10.5
Master’s	69	11.8
Do not know	9	1.5
Profession (%)		
Labourer	60	10.2
Farmer	3	0.5
Intermediate occupation	56	9.6
Employee	138	23.5
Artisan, Business owner	43	7.3
Middle manager, executive	235	40.1
Do not wish to reply	51	8.8
Household income (%)		
Less than 900 €	84	14.3
900–1499 €	131	22.4
1500–2000 €	103	17.6
2001–3000 €	96	16.4
3001–4000 €	65	11.1
More than 4000 €	60	10.2
Do not wish to respond	47	8.0
Place of origin (%)		
France	498	85.0
Europe	15	2.6
N/SS Africa	63	10.8
Asia, Americas, Oceania	10	1.7
Transmission Category (%)		
MSM	290	49.5
Heterosexual	192	32.8
IV Drug Use	66	11.3
Other	38	6.5
CDC category C (%)	117	20.1
Last CD4 cell count (cells/ml, %)		
≧ 500	422	72.0
200–499	114	19.5
< 200	15	2.6
Missing	35	6.0
Last viral load (copies/mL, %)		
< 50	543	92.7
50–200	14	2.39
≧ 200	11	1.9
Missing	18	3.1
Median time (years) since start of ART (IQR)		
	583	16.4 (8.3–21.7)

^a^Completed at least 1st item of the WHOQOL-HIV BREF questionnaire: How would you rate your quality of life?

### Score distributions

The descriptive statistics of each item and domain are presented in Table 2. The proportion of missing item-level responses ranged from 0.7–2.6%. The items with the most missing responses were “How much do you need any medical treatment to function in your daily life?” and “How satisfied are you with your personal relationships?”. All items were negatively skewed. Five of the 31 items pertaining to activities of daily living, physical environment, health and social care, transportation and forgiveness and blame were strongly skewed to the left with coefficients of less than – 1.0. Kurtosis coefficients, measuring the heaviness of the tails of the distribution, ranged between 1.87 and 4.7. Floor effects were found for one item pertaining to “personal relationships”, with 22.4% of respondents responding in the lowest category. Ceiling effects were detected in 11 out of 31 items. Overall, the spirituality and personal beliefs domain had the highest score (15.04 ± 3.35) and the psychological health domain had the lowest score (13.70 ± 2.78).

**Table 2:** Descriptive statistics of the French WHOQOL-HIV BREF (*N* = 586)

Domain or item	Item number	N	Missing (%)	Mean	SD	Skewness	Kurtosis	Floor effect (%)	Ceiling effect (%)
**Overall QOL/General Health**									
Overall Quality of life	Q_1_	586	–	3.67	0.80	− 0.45	3.43	1.0	12.6
General health perception	Q_2_	586	–	3.55	0.95	−0.74	3.15	3.1	10.8
**I. Physical health**		**574**		**14.15**	**2.96**				
Pain and discomfort^a^	Q_3_	582	0.7	3.93	1.10	−0.72	2.56	2.4	41.1
HIV symptoms^a^	Q_4_	580	1.0	3.80	1.14	−0.56	2.31	3.0	36.1
Energy and fatigue	Q_14_	582	0.7	3.35	0.89	−0.36	3.00	2.8	7.1
Sleep and rest	Q_14_	578	1.4	3.06	1.17	−0.35	2.11	13.6	7.3
**II. Psychological health**		**569**		**13.70**	**2.78**				
Positive feelings	Q_6_	581	0.9	3.33	0.96	−0.51	3.19	5.7	8.7
Concentration	Q_11_	577	1.5	3.80	0.92	−0.38	2.87	1.7	14.9
Bodily image and appearance	Q_15_	582	0.7	3.13	1.07	−0.33	2.69	10.2	9.0
Self-esteem	Q_24_	577	1.5	3.52	0.93	−0.67	3.39	3.6	10.8
Negative feelings^a^	Q_31_	578	1.4	3.54	1.00	−0.62	3.10	4.3	14.4
**III. Level of independence**		**560**		**14.65**	**3.49**				
Dependence on medication or treatment^a^	Q_5_	571	2.6	3.32	1.55	−0.22	1.49	17.4	37.5
Activities of daily living	Q_20_	583	0.5	4.14	0.97	−1.13	3.83	1.9	44.3
Work capacity	Q_22_	579	1.2	3.69	0.94	−0.82	3.48	2.8	16.3
Mobility	Q_23_	573	2.2	3.49	1.14	−0.76	2.82	9.1	15.6
**IV. Social relations**		**557**		**13.91**	**3.03**				
Social support	Q_17_	577	1.5	3.85	0.96	−0.96	3.90	3.1	24.1
Sexual activity	Q_25_	577	1.5	3.67	0.91	−0.96	4.06	3.5	12.9
Personal relationships	Q_26_	571	2.6	2.77	1.25	−0.02	1.87	22.4	7.2
Social inclusion	Q_27_	574	2.0	3.64	0.99	−0.79	3.60	4.9	17.0
**V. Environmental health**		**557**		**14.37**	**2.57**				
Physical safety and security	Q_12_	582	0.7	3.51	0.99	−0.79	3.52	6.4	12.0
Home environment	Q_13_	582	0.7	3.53	1.14	−0.84	3.06	9.6	16.8
Financial resources	Q_16_	577	1.5	2.90	1.15	−0.16	2.35	16.8	7.8
Opportunities to acquire new skills and information	Q_18_	580	1.0	3.65	0.94	−0.79	3.53	3.1	14.5
Participation in and opportunities for recreation and leisure activities	Q_19_	578	1.4	3.02	1.15	−0.27	2.27	13.8	7.8
Physical environment	Q_28_	577	1.5	3.98	0.96	−1.20	4.56	3.3	30.3
Health and social care	Q_29_	576	1.7	4.16	0.83	−1.18	4.72	0.9	36.6
Transport	Q_30_	576	1.7	3.95	0.96	−1.11	4.33	3.3	29.0
**VI. Spirituality/religion and personal beliefs**		**570**		**15.04**	**3.35**				
Religion, spirituality and personal beliefs	Q_7_	580	1.0	3.35	1.18	−0.51	2.44	10.0	15.2
Forgiveness and blame^a^	Q_8_	580	1.0	4.28	1.19	−1.47	3.88	4.3	67.1
Concerns about the future^a^	Q_9_	580	1.0	3.62	1.23	−0.58	2.36	7.1	29.1
Death and dying^a^	Q_10_	578	1.4	3.80	1.24	−0.74	2.50	6.4	39.3

^a^Reversed items recoded

### Reliability

Four of the six domains showed good internal consistency (Cronbach’s α ranged from 0.63 to 0.79) (Table 3). The physical health and the spirituality domains had a Cronbach’s α of 0.63 and 0.64 respectively, which are somewhat below the threshold of 0.70 for acceptable internal consistency.

**Table 3:** Internal consistency of the WHOQOL-HIV BREF

Domain	N	Mean	SD	Range		Cronbach’s α Coeff.
				Min	Max	
I. Physical health	574	14.15	2.97	4	20	0.63
II. Psychological health	569	13.70	2.78	4.8	20	0.76
III. Level of independence	560	14.65	3.49	4	20	0.72
IV. Social relations	557	13.91	3.03	4	20	0.70
V. Environmental health	557	14.37	2.57	6	20	0.79
VI. Spirituality/religion and personal beliefs	570	15.04	3.35	4	20	0.64

### Criterion validity

#### Concurrent validity

The correlation coefficients of all domains with the two general measures (general QoL and health perception) for each of the six domains is presented in Table 4. All domains correlated with both general quality of life (How would you rate your quality of life?) and general health perception (How satisfied are you with your health?) significantly (*p* < 0.001). With the exception of the domain pertaining to spirituality and personal beliefs, the correlation coefficients were greater than 0.40 (range of r = 0.44–0.59) for domains and general perception of quality of life. Correlations between domains and general health perception were weaker, with correlation coefficients ranging from 0.33–0.47. Physical and psychological health correlated more strongly with general health perception than other domains (Table 4).

**Table 4:** Concurrent validity of the WHOQOL-HIV BREF

Domain	N	Correlation coefficient			
		General QoL		General health perception	
I. Physical health	574	0.54	***	0.47	***
II. Psychological health	569	0.59	***	0.47	***
III. Level of independance	560	0.51	***	0.44	***
IV. Social relations	557	0.44	***	0.33	***
V. Environmental health	557	0.57	***	0.39	***
VI. Spirituality/religion and personal beliefs	570	0.38	***	0.33	***

*** *p* < 0.001, results of Pearson’s correlations

### Construct validity

**Fig. 1:**
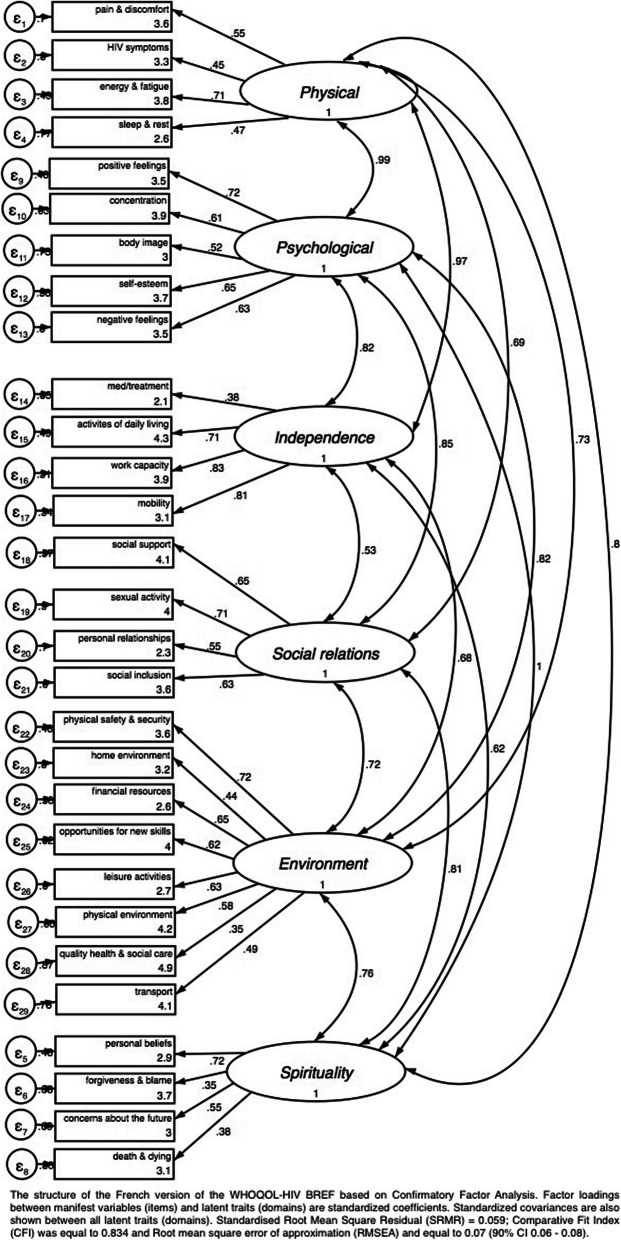
The original six-domain structure of the French version of the WHOQOL-HIV BREF based on CFA

The CFA results (Fig. 1) showed that the six-domain structure of the WHOQOL-HIV BREF produced an acceptable fit to the data (SRMR = 0.059; CFI = 0.834; RMSEA = 0.070; 90% CI: 0.066–0.075). The factor loading of each item with its respective domain was acceptable, ranging from 0.35 to 0.83 (Fig. 1). By including error covariances between certain facets within the environmental and spirituality domains, specifically financial resources (Q16) and leisure activities (Q19), quality of health and social care (Q29) and transport (Q10), forgiveness and blame (Q8) and concerns about the future (Q9), and finally, concerns about the future (Q9) and death and dying (Q10), we were able to improve the fit of the six-domain structure to the data (SRMR = 0.053; CFI = 0.882: RMSEA = 0.060 90% CI: 0.056–0.064).

#### Convergent and discriminant validity

Items were mostly strongly correlated with their respective domains, with correlation coefficients ranging from 0.45 to 0.82 (Table 5). All but one item were more highly correlated with their respective domains than other domains; the item regarding the spiritual domain (Question n°7: To what extent do you feel your life to be meaningful?) showed a higher correlation with the psychological domain (r = 0.67) than with the personal beliefs and spirituality domain (r = 0.47). Otherwise, convergent and discriminant validity were considered to be good (Table 6).

**Table 5:** Item-domain Pearson's correlations for the French WHOQOL-HIV BREF questionnaire

	I. Physical health	II. Psychological health	III. Level of independence	IV. Social relations	V. Environmental health	VI. Spirituality/religion and personal beliefs
Q_3_*	**0.72**	0.41	0.56	0.28	0.35	0.31
Q_4_*	**0.69**	0.43	0.36	0.36	0.31	0.39
Q_14_	**0.71**	0.64	0.59	0.43	0.46	0.39
Q_14_	**0.64**	0.45	0.41	0.27	0.26	0.30
Q_6_	0.55	**0.74**	0.49	0.50	0.58	0.50
Q_11_	0.43	**0.69**	0.45	0.44	0.50	0.40
Q_15_	0.45	**0.68**	0.39	0.39	0.35	0.34
Q_24_	0.50	**0.77**	0.53	0.50	0.37	0.42
Q_31_*	0.53	**0.70**	0.41	0.48	0.36	0.57
Q_5_*	0.37	0.32	**0.70**	0.23	0.23	0.25
Q_20_	0.56	0.46	**0.75**	0.26	0.48	0.24
Q_22_	0.62	0.59	**0.79**	0.37	0.48	0.37
Q_23_	0.60	0.60	**0.80**	0.42	0.48	0.36
Q_17_	0.34	0.43	0.29	**0.72**	0.49	0.42
Q_25_	0.38	0.57	0.34	**0.76**	0.37	0.40
Q_26_	0.36	0.50	0.32	**0.72**	0.35	0.35
Q_27_	0.32	0.41	0.28	**0.75**	0.42	0.36
Q_12_	0.48	0.63	0.47	0.49	**0.70**	0.49
Q_13_	0.19	0.26	0.17	0.21	**0.59**	0.19
Q_16_	0.35	0.42	0.40	0.36	**0.71**	0.30
Q_18_	0.33	0.41	0.33	0.43	**0.66**	0.37
Q_19_	0.38	0.45	0.42	0.36	**0.69**	0.31
Q_28_	0.28	0.38	0.30	0.41	**0.66**	0.33
Q_29_	0.23	0.22	0.23	0.25	**0.47**	0.14
Q_30_	0.26	0.27	0.34	0.30	**0.61**	0.19
Q_7_	0.41	0.67	0.41	0.50	0.48	**0.59**
Q_8_*	0.29	0.28	0.21	0.28	0.26	**0.63**
Q_9_*	0.45	0.48	0.35	0.38	0.34	**0.83**
Q_10_*	0.26	0.31	0.15	0.27	0.20	**0.74**

**Table 6:** Convergent & discriminant validity of the WHOQOL-HIV BREF

Domain	Range correlation coefficient				Convergent Validity		Discriminant validity	
	convergent validity		discriminant validity		Success^a^/Total	%	Success^b^/Total	%
	min	max	min	max				
I. Physical health	0.65	0.72	0.27	0.65	4/4	100	4/4	100
II. Psychological health	0.67	0.77	0.31	0.56	5/5	100	5/5	100
III. Level of independence	0.70	0.80	0.21	0.63	4/4	100	4/4	100
IV. Social relations	0.73	0.75	0.28	0.57	4/4	100	4/4	100
V. Environmental health	0.45	0.71	0.12	0.61	8/8	100	8/8	100
VI. Spirituality/religion and personal beliefs	0.58	0.82	0.15	0.66	4/4	100	3/4	75

^a^Success: a correlation coefficient ≧ 0.4 for items and their respective domain

^b^Success: a correlation coefficient for item greater in respective domain compared to other 5 domains

#### Known-group validity

The WHOQOL-HIV-BREF was not able to discriminate based on immunological and virological status (results not shown). We explored known-group validity according to CDC defined clinical categories for HIV infection. Overall quality of life, general health perception and domain scores were higher for those classified in clinical category A compared to clinical category B. However, no differences were detected between categories B and C (Table 7). Those who had been hospitalized for more than 48 h within 2 years of their most recent consultation had poorer overall quality of life and poorer general health perception compared to those who had not. They also reported significantly lower mean scores for the physical, psychological, level of independence and environmental health domains. However, there was no evidence of a differencein mean scores for the social and personal beliefs domains (Table 8).

**Table 7:** Known-group validity of the WHOQOL-HIV BREF instrument according to CDC clinical categories for HIV infection

	N	A			N	B			N	C			*F*
		*N* = 330				*N* = 136				*N* = 117			
Overall QoL and Health		Mean	95% CI			Mean	95% CI			Mean	95% CI		
General QoL^a^ (*N* = 586)	333	3.78	3.70	3.86	136	3.57	3.44	3.71	117	3.50	3.35	3.64	7.09**
General Health Perception^a^ (*N* = 586)	333	3.67	3.57	3.76	136	3.36	3.19	3.53	117	3.43	3.25	3.60	6.46**
**Domain**													
I. Physical health^a^ (*N* = 574)	326	14.55	14.25	14.86	135	13.61	13.09	14.14	113	13.63	13.06	14.20	7.11**
II. Psychological health^a^ (*N* = 569)	324	13.98	13.67	14.28	132	13.21	12.73	13.68	113	13.51	12.99	14.02	3.98*
III. Level of independence^a^ (*N* = 560)	317	15.18	14.82	15.55	131	13.92	13.26	14.57	112	14.00	13.38	14.62	8.73**
IV. Social relations^a^ (*N* = 557)	316	14.12	13.78	14.45	131	13.43	12.89	13.96	110	13.88	13.35	14.41	2.42
V. Environmental health^a^ (*N* = 557)	318	14.68	14.42	14.94	130	14.05	13.57	14.52	109	13.86	13.33	14.39	5.62**
VI. Spirituality/religion and personal beliefs^a^ (*N* = 570)	325	15.30	14.95	15.66	132	14.53	13.95	15.11	113	14.90	14.26	15.55	2.63

ANOVA; * *p* < 0.01 ** *p* < 0.001

^a^Category A is significantly different than category B based on T-test; No differences detected between B and C categories

**Table 8:** Comparison of mean scores among those who were hospitalized for > 48 h (*n* = 31) vs. those who were not (*n* = 555)

		Not hospitalized > 48 h				Hospitalization > 48 h (within last 2 years)				
	N	N	Mean	95% CI		N	Mean	95% CI		T-value^a^
Overall QoL and Health				l.b.	u.b			l.b.	u.b	
General QoL	586	555	3.71	3.64	3.77	31	3.13	2.77	3.49	3.97****
General Health	586	555	3.58	3.50	3.66	31	3.03	2.69	3.38	3.16***
Domain										
I. Physical health	574	544	14.24	13.99	14.49	30	12.73	11.72	13.74	2.71**
II. Psychological health	568	538	13.76	13.53	14.00	30	12.56	11.66	13.46	2.31*
III. Level of independence	560	532	14.80	14.51	15.08	28	11.93	10.43	13.43	4.31****
IV. Social relations	556	526	13.93	13.67	14.19	30	13.43	12.48	14.38	0.87
V. Environmental health	557	531	14.43	14.21	14.65	26	13.21	12.15	14.27	2.36**
VI. Spirituality/religion and personal beliefs	570	540	15.05	14.77	15.34	30	14.77	13.68	15.85	0.45

^a^T-test, T-Value;*p < 0.05 ** *p* < 0.01; *** *p* < 0.001; **** *p* < 0.0001

## Discussion

The French version of the WHOQOL-HIV BREF presented good cross-cultural relevance and acceptable measurement properties in a sample of PLWH who are community-dwelling and mostly virally suppressed. We did, however, observe ceiling effects for a number of items. Some of these are expected, given our sample’s clinical characteristics and the current standard of HIV care in [south-western] France. For example, ceiling effects were observed for physical pain and HIV symptoms, with 41.1% responding that they were not at all hampered by physical pain and 36.1% stating that they were not at all bothered by physical problems related to HIV. The physical health domain comprised of only four items, including these two, may therefore fail to discriminate among subjects at the top end of the scale. We observed the greatest ceiling effect for the item pertaining to forgiveness and blame (To what extent are you bothered by people blaming you for your HIV status?), with 67.1% responding that they were not at all bothered. This may be a reflection of the absence of guilt in the majority of our sample, one keen to actively participate in research related to their HIV infection outside of the hospital setting.

CFA suggested acceptable fit to our data. The SRMR suggested good model fit. CFI, which compares the fit of a target model to the fit of an independent or null model, and RMSEA, measuring the discrepancy between the observed and model-implied covariance matrices, adjusted for degrees of freedom, suggested acceptable model fit. However, we observed that the addition of error covariances between items improved the model’s fit, albeit marginally. All the first-order factor loadings were moderate to high. We, therefore, do not recommend that these items be removed from the WHOQOL-HIV BREF questionnaire. Nevertheless, one item from the spiritual health domain appeared to be better correlated with the psychological health domain.

Somewhat unsurprisingly given the clinical presentation of those in our sample, the WHOQOL-HIV BREF questionnaire was neither able to discriminate between CD4 cell count groups nor between those who had or had not achieved viral suppression (most recent measurement within 3 years of the last consultation). One reason for this finding may be the fact that 99.5% of the participants were on ART and only 2.6% of the participants in the current study were significantly immunosuppressed, with CD4 cell counts below 200 cells/mm3, and only 7.0% had a detectable viral load, defined as greater than 50 copies copies/mL. Nevertheless, there was some evidence of a difference in both general items and domain scores between CDC clinical category A compared to those in clinical category B. However, we were not able to detect a difference between categories B and C. Immune restoration as a result of ART provides some explanation for the absence of differences between categories B and C [32].

Previous studies have been conducted to assess the validity of Portuguese [33], Spanish [34], Finnish [35], Chinese [18], Malay [19], Taiwanese [36], Persian [37], and Thai [38] versions of the WHOQOL-HIV BREF. Our findings regarding the WHOQOL-HIV BREF’s less than ideal internal consistancy are similar to those of Nobre [35], Hsiung [36], Zhu [18], Meemon [38] and Fuster-Ruizde Apodaca [34] who also reported lower internal consistency in the physical health and spirituality/personal beliefs domains compared to the other four domains. With regards to the instrument’s ability to discriminate between known-groups, specifically those based on CD4 cell count thresholds, findings have been mixed. Some have reported that WHOQOL-HIV BREF detected differences between CD4 cell count groups [18, 34]; while others like Nobre et al., in a population quite similar to ours in Finland, and Meemon, in a population where only 11.6% had advanced disease, have not been able to detect differences [35, 38]. While CD4 cell count monitoring has historically been used for the assessment of disease progression and the appropriate management of patients with advanced disease [39], its value in the current and future treatment era, one in which the vast majority of PLWH are stable on ART, is currently being questioned [13].

The WHOQOL-HIV BREF was developed in an effort to overcome the main limitation of the WHOQOL-HIV: its length. It was not, however, developed for clinical research or care but rather as means to assess the impact of large-scale interventions on the multi-dimensional QoL of PLWH and to monitor QoL in PLWH across different countries. The instrument’s inability to detect differences between clinically meaningful thresholds of immunological and virological status may make its usefulness for clinical research limited, especially in those who are asymptomatic. However, the WHOQOL-HIV BREF does appear to be sensitive to known disease groups, specifically those who have experienced symptomatic-HIV or AIDS or been recently hospitalized and thus continues to be valuable for population health. Instruments with a broader scope, like the WHOQOL-HIV BREF, may also aid clinicians who seek to account for or address indirect determinants of individual health outcomes (e.g. social isolation or housing) [40, 41]. To date, due to the limited number of longitudinal studies on QoL in the current treatment era [42], there is still limited evidence regarding the WHOQOL-HIV BREF’s responsiveness to within patient changes over time. Furthermore, within patient changes in QoL may not necessarily be related to clinical manifestations of HIV infection but rather to associated comorbidities [4].

### Strengths & Limitations

Given the effort, money and time required to develop a new instrument designed to measure a multi-dimensional construct like (HR)QoL, many have urged researchers to rely on existing instruments and ensure their validity in new populations. We have followed this recommendation. This study has the advantage of drawing on objective and detailed clinical and laboratory data which were prospectively collected within the ANRS CO3 Aquitaine cohort. However, we enrolled PLWH on a voluntary basis in clinic and relied on their willingness and ability to complete a self-administered assessment. This resulted in the exclusion of people who had severe neurocognitive impairment or were not able to understand and/or read French sufficiently well. This recruitment strategy may have resulted in a less representative sample of French-speaking people in care in 2019.

This analysis relies on a classical test theory (CTT) rather than item response theory measurement framework as our intial goal was to ensure that the French version, which has been previously validated using CTT, had acceptable psychometric properties in our population. Unfortunately, we could not assess test-retest reliability as only one time point was available at the time of this analysis. Zhu et al. has nevertheless reported good to excellent reliability in an assessment repeated at 2 weeks in 57 patients. The intraclass correlation coefficients for the six dimensions ranged from 0.72–0.82, with coefficients of greater or equal to 0.70 being commonly accepted as adequate [18]. We did not explore measurement invariance between different subgroups, for example, between men and women, as only 156 women responded. These are areas for future research.

## Conclusions

The WHOQOL-HIV BREF, going beyond physical and mental health, has acceptable measurement properties in our older, treatment-experienced and virally suppressed population. Our findings nevertheless shed light on some of its potential shortcomings, which are relevant for future research in an era where an increasing number of PLWH are doing well on ART.

## Supplementary information

**Additional file 1.**

**Additional file 2.**

## Data Availability

The datasets generated during and/or analysed during the current study are not publicly available due to ongoing work but are available from the corresponding author on reasonable request.

## Abbreviations

AIDS: Acquired Immune Deficiency Syndrome
ANOVA: Analysis of variance
ART: Antiretroviral therapy
CDC: Centers for Disease Control and Prevention
CFA: Confirmatory Factor Analysis
CFI: Comparative Fix Index
CTT: Classical Test Theory
ePRO.: Electronic patient-reported outcomes
HRQoL: Health-related quality of life
HIV: Human immunodeficiency virus
MSM: Men who have sex with men
IV: Intravenous
PRO: Patient-reported outcomes
PLWH: People living with HIV
SRMR: Standardised Root Mean Square Residual
RMSEA: The Root Mean Square Error of Approximation
QoL: Quality of Life
