# Supplementary material for: Assessing the psychometric properties of the French WHOQOL-HIV BREF within the ANRS CO3 Aquitaine Cohort’s QuAliV ancillary study
Source: Health Qual Life Outcomes. 2020 Jul 10;18:220. doi: 10.1186/s12955-020-01451-8 (PMC7350695; doi:10.1186/s12955-020-01451-8)
Supplement: Supplementary file 1 — Additional file 1. [file 12955_2020_1451_MOESM1_ESM.pdf]

WHOQOL-HIV BREF

Lisez bien les questions et choisissez le bouton qui correspond à la réponse la mieux adaptée à ce que vous ressentez au cours des **deux dernières semaines**.

		Très mauvaise	Mauvaise	Ni bonne, ni mauvaise	Bonne	Très bonne
1	Comment évaluez-vous votre qualité de vie ?	1	2	3	4	5

		Pas du tout satisfait	Plutôt pas satisfait	Ni satisfait ni satisfait	Plutôt satisfait	Très satisfait
2	Êtes-vous satisfait de votre santé ?	1	2	3	4	5

Les questions suivantes portent sur la manière dont vous avez ressenti certaines situations au cours des **deux dernières semaines**.

		Pas du tout	Un peu	Modérément	Beaucoup	Extrêmement
3	La douleur physique vous empêche-t-elle de faire ce dont vous avez envie ?	1	2	3	4	5
4	Dans quelle mesure, êtes-vous tracassé par tout problème physique lié à votre infection par le VIH ?	1	2	3	4	5
5	Un traitement médical vous est-il nécessaire pour faire face à la vie de tous les jours ?	1	2	3	4	5
6	Trouvez-vous la vie agréable ?	1	2	3	4	5
7	Estimez-vous que votre vie a du sens ?	1	2	3	4	5
8	Êtes-vous affecté par les reproches des personnes autour de vous par rapport au VIH ?	1	2	3	4	5
9	Avez-vous peur de l'avenir ?	1	2	3	4	5
10	Êtes-vous inquiet par rapport à la mort ?	1	2	3	4	5
11	Êtes-vous capable de vous concentrer ?	1	2	3	4	5
12	Vous sentez-vous en sécurité dans votre vie de tous les jours ?	1	2	3	4	5
13	Vivez-vous dans un environnement sain (pollution, bruit, salubrité, ...) ?	1	2	3	4	5

Les questions suivantes visent à évaluer dans quelle mesure vous avez vécu ou été capable de faire certaines choses de manière complète au cours des **deux dernières semaines.**

		Pas du tout	Un peu	Modérément	Beaucoup	Extrêmement
14	Avez-vous assez d'énergie dans la vie de tous les jours ?	1	2	3	4	5
15	Acceptez-vous votre apparence physique ?	1	2	3	4	5
16	Avez-vous assez d'argent pour satisfaire vos besoins ?	1	2	3	4	5
17	Vous sentez-vous accepté par votre entourage ?	1	2	3	4	5

18	Avez-vous le sentiment d'être assez informé pour faire face à la vie de tous les jours ?	1	2	3	4	5
19	Avez-vous l'occasion de pratiquer des loisirs ?	1	2	3	4	5

		Très mal	Mal	Ni mal, ni bien	Bien	Très bien
20	Comment arrivez-vous à vous déplacer ?	1	2	3	4	5

Les questions suivantes portent sur la manière dont vous avez été satisfait, heureux ou bien par rapport aux différents aspects de votre vie au cours des **deux dernières semaines**.

		Pas du tout satisfait	Plutôt pas satisfait	Ni satisfait ni insatisfait	Plutôt satisfait	Très satisfait
21	Êtes-vous satisfait de votre sommeil ?	1	2	3	4	5
22	Êtes-vous satisfait de votre capacité à effectuer les tâches de la vie quotidienne ?	1	2	3	4	5
23	Êtes-vous satisfait de votre capacité à travailler ?	1	2	3	4	5
24	Êtes-vous satisfait de vous ?	1	2	3	4	5
25	Êtes-vous satisfait de vos relations avec les autres ?	1	2	3	4	5
26	Êtes-vous satisfait de votre vie sexuelle ?	1	2	3	4	5
27	Êtes-vous satisfait du soutien de vos amis ?	1	2	3	4	5
28	Êtes-vous satisfait avec votre lieu de vie ?	1	2	3	4	5
29	Êtes-vous satisfait de l'accès aux services de santé ?	1	2	3	4	5
30	Êtes-vous satisfait de vos moyens de transport ?	1	2	3	4	5

La question suivante se rapporte à la fréquence avec laquelle vous avez ressenti ou vécu certaines choses pendant les **deux dernières semaines**.

		Jamais	Parfois	Assez Souvent	Très souvent	Tout le temps
31	Avez-vous des sentiments négatifs tels que de la mélancolie, du désespoir, de l'anxiété, de la dépression ?	1	2	3	4	5
