# Supplementary material for: Assessing the psychometric properties of the French WHOQOL-HIV BREF within the ANRS CO3 Aquitaine Cohort’s QuAliV ancillary study
Source: Health Qual Life Outcomes. 2020 Jul 10;18:220. doi: 10.1186/s12955-020-01451-8 (PMC7350695; doi:10.1186/s12955-020-01451-8)
Supplement: Supplementary file 2 — Additional file 2. [file 12955_2020_1451_MOESM2_ESM.pdf]

Table A:1. Pearson's correlations for the WHOQOL-HIV BREF questionnaire, from 511 people living with HIV in the Aquitaine cohort

	I. Physical health				II. Psychological health					III. Level of independence				IV. Social relations				V. Environmental health								VI. Spirituality/religion and personal beliefs			
	Q_3*	Q_4*	Q_{14}	Q_{14}	Q_6	Q_{11}	Q_{15}	Q_{24}	Q_{31}*	Q_5*	Q_{20}	Q_{22}	Q_{23}	Q_{17}	Q_{25}	Q_{26}	Q_{27}	Q_{12}	Q_{13}	Q_{16}	Q_{18}	Q_{19}	Q_{28}	Q_{29}	Q_{30}	Q_7	Q_8*	Q_9*	Q_{10}*
Q_3*	1																												
Q_4*	0.35	1																											
Q_{14}	0.37	0.36	1																										
Q_{14}	0.24	0.15	0.32	1																									
Q_6	0.37	0.36	0.47	0.32	1																								
Q_{11}	0.27	0.20	0.48	0.27	0.42	1																							
Q_{15}	0.23	0.28	0.44	0.32	0.36	0.33	1																						
Q_{24}	0.27	0.26	0.50	0.37	0.46	0.43	0.43	1																					
Q_{31}*	0.31	0.41	0.41	0.33	0.44	0.33	0.28	0.46	1																				
Q_5*	0.31	0.22	0.26	0.22	0.23	0.17	0.19	0.28	0.27	1																			
Q_{20}	0.54	0.24	0.48	0.29	0.43	0.37	0.30	0.31	0.22	0.29	1																		
Q_{22}	0.44	0.29	0.57	0.41	0.46	0.42	0.34	0.52	0.37	0.28	0.58	1																	
Q_{23}	0.46	0.37	0.52	0.32	0.45	0.44	0.36	0.52	0.38	0.28	0.57	0.71	1																
Q_{17}	0.22	0.27	0.34	0.14	0.37	0.36	0.27	0.28	0.28	0.13	0.23	0.23	0.29	1															
Q_{25}	0.17	0.27	0.39	0.24	0.40	0.35	0.29	0.53	0.46	0.17	0.19	0.33	0.36	0.46	1														
Q_{26}	0.21	0.26	0.30	0.24	0.39	0.30	0.33	0.41	0.37	0.20	0.18	0.26	0.31	0.28	0.38	1													
Q_{27}	0.23	0.25	0.27	0.16	0.32	0.30	0.27	0.26	0.30	0.18	0.16	0.24	0.26	0.47	0.46	0.33	1												

Table A.2 – Pearson's correlations for the WHOQOL-HIV BREF questionnaire, from 511 people living with HIV in the Aquitaine cohort (Continued)

	I. Physical health				II. Psychological health					III. Level of independence				IV. Social relations				V. Environmental health								VI. Spirituality/religion and personal beliefs			
	Q_{3}*	Q_{4}*	Q_{14}	Q_{14}	Q_{6}	Q_{11}	Q_{15}	Q_{24}	Q_{31}*	Q_{5}*	Q_{20}	Q_{22}	Q_{23}	Q_{17}	Q_{25}	Q_{26}	Q_{27}	Q_{12}	Q_{13}	Q_{16}	Q_{18}	Q_{19}	Q_{28}	Q_{29}	Q_{30}	Q_{7}	Q_{8}*	Q_{9}*	Q_{10}*
Q_{12}	0.30	0.32	0.44	0.27	0.55	0.55	0.31	0.41	0.44	0.25	0.36	0.41	0.43	0.38	0.37	0.30	0.40	1											
Q_{13}	0.10	0.11	0.23	0.12	0.23	0.26	0.18	0.15	0.14	0.06	0.15	0.14	0.17	0.21	0.14	0.14	0.15	0.34	1										
Q_{16}	0.30	0.22	0.32	0.15	0.46	0.33	0.27	0.20	0.23	0.15	0.40	0.34	0.37	0.39	0.17	0.27	0.25	0.43	0.32	1									
Q_{18}	0.24	0.23	0.30	0.16	0.37	0.36	0.24	0.26	0.26	0.13	0.27	0.33	0.29	0.44	0.27	0.25	0.35	0.42	0.25	0.39	1								
Q_{19}	0.31	0.19	0.38	0.17	0.50	0.34	0.26	0.25	0.25	0.20	0.44	0.34	0.38	0.32	0.23	0.27	0.24	0.42	0.32	0.55	0.38	1							
Q_{28}	0.18	0.19	0.27	0.16	0.35	0.36	0.18	0.27	0.24	0.16	0.26	0.28	0.24	0.32	0.33	0.24	0.35	0.40	0.37	0.37	0.39	0.32	1						
Q_{29}	0.17	0.17	0.17	0.14	0.20	0.15	0.15	0.19	0.10	0.06	0.19	0.25	0.22	0.19	0.20	0.15	0.21	0.22	0.10	0.13	0.29	0.17	0.28	1					
Q_{30}	0.20	0.17	0.20	0.14	0.25	0.20	0.16	0.21	0.16	0.14	0.34	0.34	0.30	0.23	0.21	0.18	0.25	0.33	0.22	0.31	0.32	0.26	0.30	0.42	1				
Q_{7}	0.24	0.25	0.42	0.24	0.65	0.45	0.38	0.47	0.47	0.22	0.27	0.36	0.40	0.36	0.42	0.34	0.38	0.50	0.21	0.32	0.39	0.40	0.32	0.14	0.16	1			
Q_{8}*	0.19	0.24	0.19	0.17	0.22	0.20	0.14	0.17	0.26	0.17	0.13	0.16	0.14	0.28	0.19	0.20	0.17	0.21	0.12	0.24	0.21	0.17	0.20	0.06	0.10	0.16	1		
Q_{9}*	0.30	0.37	0.32	0.25	0.34	0.29	0.24	0.34	0.50	0.20	0.20	0.33	0.32	0.31	0.29	0.27	0.27	0.39	0.10	0.20	0.30	0.19	0.24	0.15	0.16	0.33	0.36	1	
Q_{10}*	0.12	0.22	0.17	0.18	0.19	0.21	0.18	0.20	0.35	0.11	0.05	0.16	0.11	0.21	0.21	0.18	0.21	0.26	0.10	0.11	0.14	0.12	0.17	0.05	0.09	0.19	0.25	0.60	1
